# Embedding Ultrafine and High‐Content Pt Nanoparticles at Ceria Surface for Enhanced Thermal Stability

**DOI:** 10.1002/advs.201700056

**Published:** 2017-05-04

**Authors:** Jingshan S. Du, Ting Bian, Junjie Yu, Yingying Jiang, Xiaowei Wang, Yucong Yan, Yi Jiang, Chuanhong Jin, Hui Zhang, Deren Yang

**Affiliations:** ^1^ State Key Laboratory of Silicon Materials School of Materials Science and Engineering Zhejiang University Hangzhou 310027 P. R. China; ^2^ School of Energy and Power Engineering Jiangsu University of Science and Technology Zhenjiang 212003 P. R. China

**Keywords:** embedded structures, in situ study, Pt/CeO_2_ catalysts, sinter resistance, thermal stability

## Abstract

Ultrafine Pt nanoparticles loaded on ceria (CeO_2_) are promising nanostructured catalysts for many important reactions. However, such catalysts often suffer from thermal instability due to coarsening of Pt nanoparticles at elevated temperatures, especially for those with high Pt loading, which leads to severe deterioration of catalytic performances. Here, a facile strategy is developed to improve the thermal stability of ultrafine (1–2 nm)‐Pt/CeO_2_ catalysts with high Pt content (≈14 wt%) by partially embedding Pt nanoparticles at the surface of CeO_2_ through the redox reaction at the solid–solution interface. Ex situ heating studies demonstrate the significant increase in thermal stability of such embedded nanostructures compared to the conventional loaded catalysts. The microscopic pathways for interparticle coarsening of Pt embedded or loaded on CeO_2_ are further investigated by in situ electron microscopy at elevated temperatures. Their morphology and size evolution with heating temperature indicate that migration and coalescence of Pt nanoparticles are remarkably suppressed in the embedded structure up to about 450 °C, which may account for the improved thermal stability compared to the conventional loaded structure.

## 1 Introduction

Noble‐metal catalysts, such as Pt‐based nanostructures, play a crucial role in many important industrial processes due to their fascinating catalytic properties. However, the whole industry and research efforts have been fighting with the sintering effect of the highly active nanoparticles (NPs) for more than decades because aggregation and coarsening significantly lowers the active area of catalysts. It is therefore necessary to load or anchor metal NPs on thermally stable supports in an aim to physically separate and stabilize the active sites.^1^ Among the various types of materials, ceria (CeO_2_) has been particularly recognized as a very important support for Pt due to its high oxygen storage capacity^2^ and the strong metal–support interaction (SMSI) effect between them.^3^, ^4^ As such, loading Pt NPs to nanostructured CeO_2_ has attracted considerable research interests in the past decades, including the approaches in the organic phase,^5^ aqueous solution routes,^6^, ^7^, ^8^ and vapor depositions.^9^, ^10^

Because of the requirement of high temperature for both catalytic reactions and catalyst activation by heat treatments, sintering of noble‐metal NPs in these scenarios is not well avoided,^1^ especially when highly active ultrafine NPs are used. Three major approaches have been exploited to enhance the thermal stability of supported metal NPs with the aid of a third material for encapsulation or as a template. To this end, Yan and co‐workers reported the microemulsion‐mediated synthesis of Pt/CeO_2_@SiO_2_ nanocomposites with removable silica shells, which protected Pt NPs from coalescence during the precalcination.^5^ The coalescence was inhibited to a certain degree, but the final product after removing silica shells still contained large Pt NPs with various sizes. Silica‐encapsulated Pt/support nanostructures were also directly applied as a catalyst with enhanced catalytic properties in the work of Xia and co‐workers,^11^ which was further improved by partially covering Pt NP “islands” in a “sea” of silica layer in a later report.^12^ A sacrificial template‐mediated method was also developed to stabilize Pt NPs. In this synthesis, Pt NPs were first loaded onto a template, followed by the encapsulation of the actual support material. After the removal of the template, Pt NPs were embedded at the inner surface of the support.^13^, ^14^

Recently, tremendous efforts have been devoted to stabilizing noble‐metal NPs with the support directly,^15^ without introducing a third‐party material for either protection or acting as a sacrificial template. For this purpose, various structures, such as core–shell,^8^, ^16^, ^17^, ^18^, ^19^, ^20^, ^21^ yolk–shell,^16^, ^22^ or hollow core–shell,^23^ were successfully generated, in which noble‐metal NPs were completely encapsulated by the support (e.g., CeO_2_). However, the concern for such encapsulated structures may fall in the sluggish kinetics of reagents reaching the active catalytic sites, since noble metals like Pt as well as the Pt–CeO_2_ interfaces are generally considered as the active sites in many important reactions.^4^, ^24^ As such, constructing a thermally stable Pt/CeO_2_ hybrid catalyst without compromising the catalytic activity is still an open challenge to the community.

Here, we demonstrate a facile approach to partially embed Pt NPs at the surface of CeO_2_ nanorods by the in situ redox reaction between Ce^III^ and Pt^II^ ions at the solid–solution (S–S) interface. Ex situ heating experiment and in situ electron microscopy studies at varied temperatures both indicate the substantially improved thermal stability of such embedded nanostructures compared to the conventional loaded ones.

## 2 Results and Discussion

**Figure 1:**
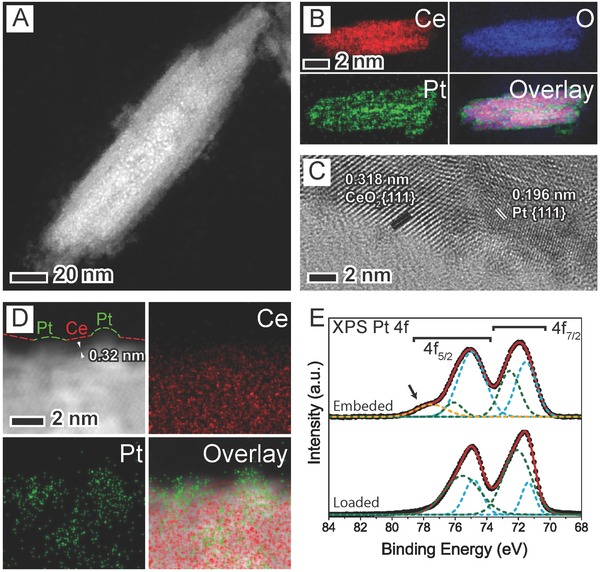
Morphological, structural, and compositional characterizations of the surface‐embedded Pt/CeO_2_ hybrid nanorods. A) HAADF‐STEM and B) HAADF‐STEM‐EDX mapping images of a single nanorod. C) HRTEM image showing lattice fringes of CeO_2_{111} and Pt{111}. D) HAADF‐STEM‐EDX mapping images at a high magnification of a local area on the nanorod showing the confinement of Pt NPs by the surrounding CeO_2_. Red and green dashes in the upper left panel are, respectively, partial profiles of CeO_2_ and Pt, showing their interlacement at the surface. E) XPS spectra of Pt 4f showing energy shift and the emergence of highly oxidized Pt species (marked by an arrow) in the surface‐embedded sample compared to that of Pt loaded on CeO_2_.

The surface‐embedded Pt/CeO_2_ hybrid nanostructures were synthesized by titrating an aqueous solution containing Ce(NO_3_)_3_ and cetyltrimethylammonium bromide (CTAB) with NaOH solution using a syringe pump at 70 °C under Ar protection, with K_2_PtCl_4_ solution being injected halfway. The hybrid nanostructure shows a rod‐like shape with dense and small NPs of 1–2 nm in size anchored on the surface, as revealed by transmission electron microscopy (TEM) (Figures S1 and S3A, Supporting Information) and aberration‐corrected high‐angle annular dark‐field scanning TEM (HAADF‐STEM) images (**Figure** **1**A). These nanorods are composed of fluorite‐type CeO_2_, as revealed by selected area electron diffraction (SAED) in Figure S2 (Supporting Information). Energy dispersive X‐ray spectroscopy (EDX) mapping and high‐resolution transmission electron microscopy (HRTEM) images indicate that Pt NPs are dispersed uniformly on the surface of CeO_2_ nanorods (Figure 1B), with the lattice fringes of Pt and CeO_2_ being clearly revealed (Figure 1C). Inductively coupled plasma mass spectrometry (ICP‐MS) shows that the Pt loading reaches ≈14 wt%, which is higher than many thermally stable Pt/CeO_2_ and other related catalysts in the literatures (see Table S1 in the Supporting Information for the comparison, most of their Pt loading values are lower than 10 wt%).

In order to distinguish the structural configuration between Pt NPs and CeO_2_ support of this sample from other designs, we synthesized conventional architectures with Pt NPs either loaded on the CeO_2_ surface (the surface‐loaded sample), or encapsulated inside CeO_2_ (the encapsulated sample) for comparison. As detailed in the Experimental Section, the surface‐loaded sample was prepared by reacting preformed ceria intermediate nanorods (ceria/cerium hydroxide nanorods) with the K_2_PtCl_4_ solution. Similar to our synthesis, this reaction has been known to load ultrafine Pt NPs on reduced oxide surfaces (Figure S3B, Supporting Information).^25^, ^26^, ^27^ The encapsulated sample was prepared by simultaneously introducing K_2_PtCl_4_ during the same time when ceria nanostructures were formed (Figure S4, Supporting Information). High‐magnification HAADF‐STEM image at the edge of the surface‐embedded structure (Figure 1D, upper‐left panel, and Figure S5 (Supporting Information)) shows alternating exposure of Pt and CeO_2_. From this image, brighter Pt NPs with higher mass thickness are embedded at the surface of CeO_2_, whose {111} lattice (≈0.32 nm) was resolved. The local configuration of Pt and Ce elements were also evidenced by the corresponding EDX mapping images as shown in the other three panels. This result suggests that Pt NPs are embedded partially at the surface of CeO_2_ rather than being encapsulated, which makes them attractive for catalytic reactions at high temperatures. Therefore, such surface‐embedded Pt/CeO_2_ hybrids are different from the conventional metal‐loaded‐on‐oxide structures reported before, where Pt NPs almost attached on top of a continuous oxide surface, even if similar S–S interfacial redox reaction was used.^25^, ^26^, ^27^ In addition, although SMSI effect may also induce partial decoration of oxide overlayers to metal NPs (e.g., Pt) through high‐temperature annealing in a reducing atmosphere, such process often coarsens metal NPs up to a large size.^28^, ^29^

X‐ray photoelectron spectroscopy (XPS) was employed to study the chemical environment of Pt, since embedding Pt NPs in an oxide may lead to additional Pt—O bonding at their interface.^30^ In the Pt 4f spectra (Figure 1E), both spin states are shifted to higher binding energy in the embedded sample, compared to the loaded one. This observation suggests that more Pt atoms are in an oxidized state relative to those in the loaded one. Significantly, there is a small additional peak to be observed in the well‐resolved spectrum for the embedded sample (marked by an arrow), which can be attributed to Pt species in a higher oxidation state (quadrivalent). The presence of this peak implies an enhanced Pt–CeO_2_ interface with a strong interaction between them, which is not observed in the loaded sample. This additional high‐energy peak has also been observed in the Pt 4f XPS spectrum for the encapsulated sample (Figure S6, Supporting Information). The signal‐to‐noise ratio was severely depressed because Pt NPs are located deeper beneath the CeO_2_ surface. Nonetheless, the presence of this additional peak strongly suggests that an enhanced interface of Pt–CeO_2_ was established due to embedding. The XPS spectra of Ce 3d and O 1s in the surface‐embedded sample (Figure S7, Supporting Information) show that the CeO_2_ support is partially reduced and rich of hydroxyl species, which is presumably related to its high defect density arising from solution synthesis.

Ex situ carbon monoxide (CO) adsorption experiment was performed to compare the Pt exposure among all three architectures. The surface‐embedded, surface‐loaded, and encapsulated samples were saturated with CO and dried on KBr substrates in a CO atmosphere. Fourier transform infrared spectroscopy (FTIR) shows characteristic absorption peaks and shoulders around 2050 cm^−1^ in all three samples (Figure S8, Supporting Information), indicative of the formation of strong Pt—CO chemisorption binding.^31^, ^32^ The normalized CO adsorption features are much less significant in the encapsulated sample, confirming its extremely low Pt exposure. Small bumps around 2160 cm^−1^ can be assigned to CO adsorption on Ce^3+^ sites,^33^ which are insignificant in all samples.

**Figure 2:**
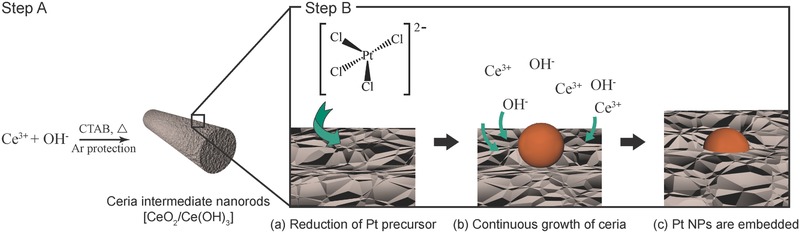
Schematic illustration for the proposed mechanism of synthesizing surface‐embedded Pt/CeO_2_ hybrid nanorods.

Our strategy to synthesize the surface‐embedded nanostructures with a rod‐like shape includes two steps in one pot, which is summarized in **Figure** **2**. The ceria/cerium hydroxide nanorods (the intermediates for the formation of CeO_2_ nanorods) were first formed by slow injection of NaOH into a Ce(NO_3_)_3_ solution due to the protection of inert gas and low temperature, as shown by Step A. The rod‐like shape was enabled by the selective adsorption of CTAB, which has been widely used to guide the synthesis of CeO_2_ nanorods and nanowires in the literatures.^34^, ^35^, ^36^ When CTAB was replaced by 6‐aminohexanoic acid or polyvinylpyrrolidone (PVP), Pt NP‐embedded nanostructures showing either a network‐like or particle‐like morphology were produced, respectively, as shown in Figure S9 (Supporting Information). This result indicates that CTAB plays a dominant role in the formation of the rod‐like shape in our synthesis.

Without incorporation of the Pt precursor, these rod‐like nanostructures (Figure S10, Supporting Information) consist of both a fluorite‐type CeO_2_ phase as well as a Ce(OH)_3_ phase with a remarkable reducing capability, as supported by the X‐ray diffraction (XRD) pattern (Figure S11, Supporting Information). In contrast, the conversion from Ce(OH)_3_ to CeO_2_ phase was performed by introducing the Pt precursor halfway during the synthesis, which resulted in the formation of the surface‐embedded Pt/CeO_2_ nanorods (Figure 2, Step B). Since no additional reducing agent was used, the formation of Pt NPs can be attributed to the solid–solution (S–S) interfacial redox reaction between Pt^II^ in the precursor (solution) and Ce^III^ in the support (solid), as illustrated by the process (a) in Step B. No Pt NPs were formed under the same conditions without cerium species. The slow reduction kinetics associated with the S–S interfacial redox reaction enables the size of Pt NPs to be restricted in an ultrafine region (1–2 nm). This S–S interfacial redox reaction is also responsible for the further oxidation of Ce(OH)_3_ to CeO_2_, which can be described by Equation (1)

(1) $${\mathrm{Pt}}^{2+}\left(\mathrm{aq}\right)\;+\;2{\mathrm{Ce}}^{3+}\left(s\right)\;\to \;{\mathrm{Pt}}^{0}\left(s\right)\;+\;2{\mathrm{Ce}}^{4+}\left(s\right)$$

With the subsequent addition of NaOH solution, the size (e.g., the average diameter and length) of the nanorods gradually increases, as evidenced by the time‐series experiment in Figure S12 (Supporting Information). This result suggests that the Pt NPs can be anchored by the newly formed CeO_2_ during the overgrowth of the nanorods, as illustrated by process (b). As such, Pt NPs are partially embedded at the surface of the CeO_2_ nanorods eventually, as shown in process (c).

The proposed synthesis is capable of tuning the Pt loading percentage on the CeO_2_ substrate. Apart from the high loading (14 wt%) sample that we have synthesized using the standard protocol, lowering Pt precursor concentration during the synthesis can result in lower Pt loading percentage. Figure S13A,B (Supporting Information) shows typical TEM images of embedded Pt/CeO_2_ samples synthesized with Pt precursor concentrations that were half or a quarter of the standard protocol. The overall morphology of these products is very similar. The average size of Pt NPs shows a slightly decreasing trend from about 1.7 nm (standard protocol) to about 1.4 nm (quarter Pt precursor concentration), although it is not possible to conclude whether this trend is statistically significant compared to the standard deviations (Figure S13C, Supporting Information). ICP‐MS reveals a positive trend of Pt loading percentage with respect to the Pt precursor concentration (Figure S13C, Supporting Information). These results demonstrated that tunable Pt loading can be achieved by using the synthesis protocol that we have developed, while the ultrafine Pt NP size is still retained.

**Figure 3:**
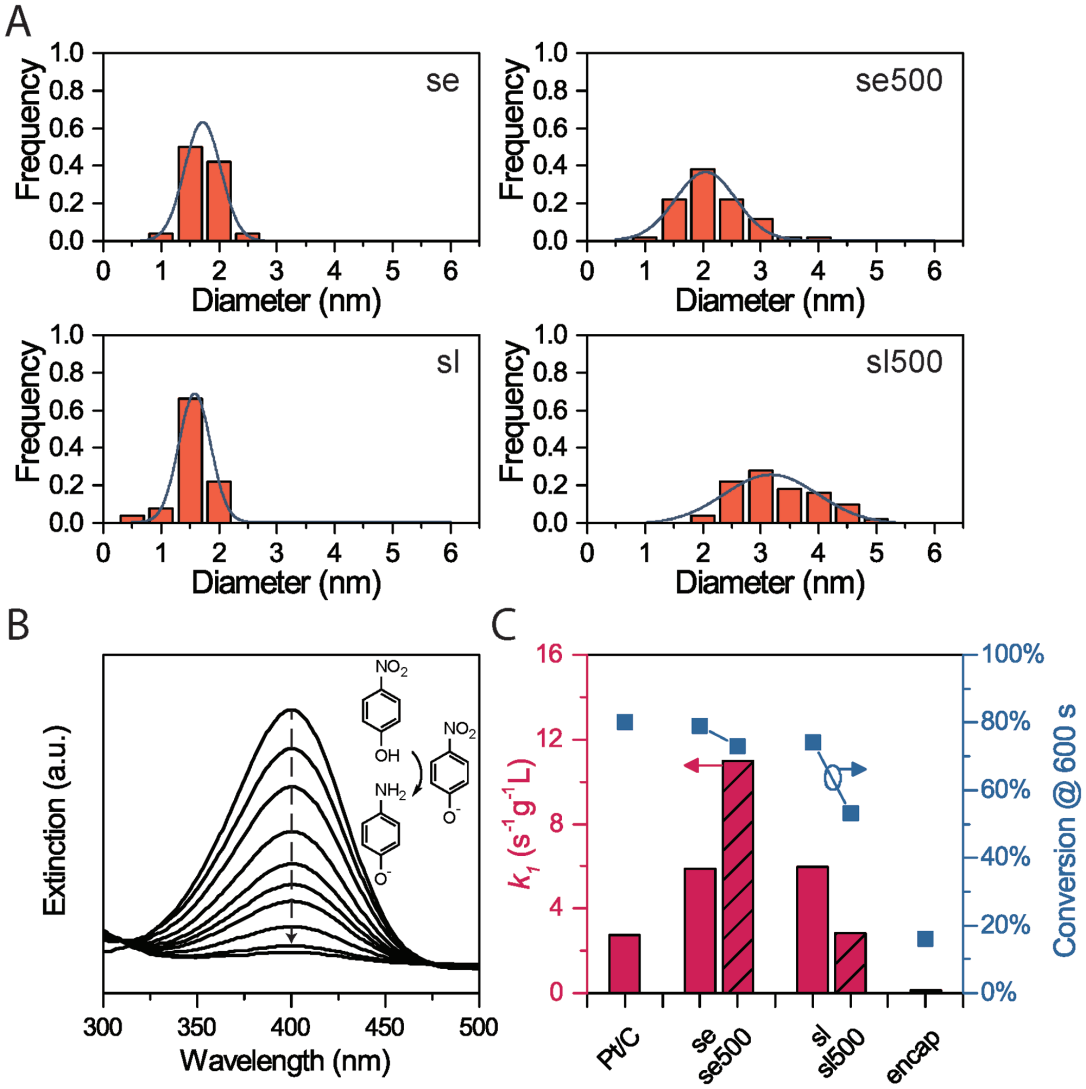
A) Size distribution of Pt NPs in surface‐embedded nanostructures before (se) and after heat treatment (se500) and in surface‐loaded nanostructures before (sl) and after heat treatment (sl500) at 500 °C. Blue curves are the corresponding Gaussian distribution fitted from the statistical data. Large Pt NPs (>10 nm) in sl500 are excluded from statistics. Embedded samples are measured using HAADF‐STEM images due to the limited contrast in TEM. B) UV–vis spectra for monitoring hydrogenation of *p*‐nitrophenol with NaBH_4_. Reaction kinetics are monitored by the decrease of the characteristic peak at 400 nm. C) First‐order Pt‐concentration‐specific rate constant (*k*_1_) and conversion efficiency at 600 s for six catalysts including the commercial Pt/C and encapsulated Pt/CeO_2_ sample.

To evaluate the thermal stability of the surface‐embedded nanostructures, we synthesized the surface‐loaded sample sharing the same Pt loading (≈14 wt%) as comparison. We first studied the size and morphology evolution of these two types of nanostructures by ex situ heating at 500 °C. The size distributions (see Table S2 in the Supporting Information) of Pt NPs were obtained from electron microscopy analyses (Figure 1A and Figures S3, S5, and S14–S16 (Supporting Information)). The fresh surface‐loaded (denoted as “sl”) and surface‐embedded (denoted as “se”) samples have similar size distributions of Pt NPs around 1–2 nm before heat treatment. In addition, the average center‐to‐center distances of Pt NPs are very similar (Table S3, Supporting Information). These results suggest that the geometrical parameters of the Pt NPs are comparable on both samples, but only the architecture of how Pt NPs are bounded to the CeO_2_ support is different. After heat treatment at 500 °C, the increase in average size and standard deviation of Pt NPs in the embedded sample (denoted as “se500”) were inhibited by about 70% and 35% compared to the surface‐loaded sample (denoted as “sl500”), as shown in **Figure** **3**A. It should be noted that large Pt aggregates (>10 nm) were generated in the surface‐loaded sample (bottom right in Figure S14B in the Supporting Information), suggesting the severe sintering effect during the heat treatment.

The hydrogenation of *p*‐nitrophenol was selected as a model reaction to evaluate the catalytic performance of the Pt/CeO_2_ hybrid samples with commercial Pt/C as a reference.^6^, ^37^, ^38^ Under the same laboratory conditions, surface‐embedded and surface‐loaded Pt/CeO_2_ catalysts with the same metal loading, or other control catalysts were, respectively, added to aqueous solutions of *p*‐nitrophenol and NaBH_4_ at a fixed molar ratio. The reaction was monitored by in situ ultraviolet‐visible (UV–vis) spectroscopy (Figure 3B), and the corresponding kinetic data are summarized in Figure 3C. We first confirmed that this catalytic hydrogenation requires the exposure of Pt rather than the other chemical species present in our catalysts. From Figure S17 (Supporting Information), the pure CeO_2_ nanorods show no catalytic activity toward the hydrogenation of *p*‐nitrophenol. This demonstration was supported by the very low catalytic activity (the “encap” sample in Figure 3C) of the Pt NPs that are largely encapsulated inside CeO_2_ nanorods (Figure S4, Supporting Information). As observed from Figure 3C, the as‐prepared surface‐embedded (se) and surface‐loaded samples (sl) have comparable activities (quantified by Pt‐concentration‐specific rate constant) and conversion efficiency. Although some Pt surface area might be sacrificed due to the partial embedding, the fact that comparable activity was achieved in the surface‐embedded sample to the loaded one suggests that the enhanced interface between Pt and CeO_2_ may further facilitated the support effect, which favors the catalytic performance of the hybrid structure. After heat treatment, both the rate constant and conversion parameters drop to nearly half of the initial value for the conventional surface‐loaded sample, which can be mainly attributed to the sintering of Pt NPs. The embedded sample, on the contrary, maintains the conversion efficiency to be still higher than 70%, while the activity increases to around 7.8 times higher than that of commercial Pt/C. Such enhancement in catalytic activity may be associated with the more optimized structure of the support, since heat treatment at 500 °C can significantly improve the crystallinity of the CeO_2_ phase (Figure S11, Supporting Information) and may also remove hydroxyl groups from the support (Figure S18, Supporting Information). These results suggest that the heat treatment functions efficiently as a catalyst activation process for the surface‐embedded sample, whereas the severe coarsening among Pt NPs may suppress its positive effect to the conventional surface‐loaded sample with a similar high loading and fine particle size of Pt NPs.

**Figure 4:**
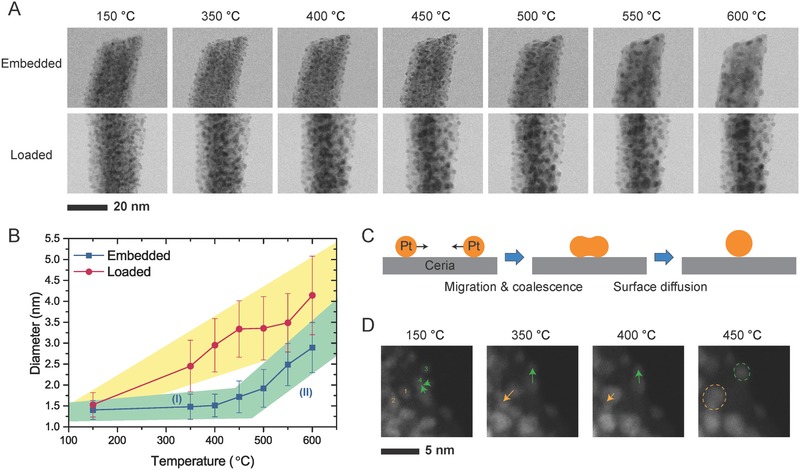
In situ heating experiment tracked by electron microscopy. A) Sequential bright field (BF) STEM images of surface‐embedded and surface‐loaded samples at different temperatures. B) Size evolution of Pt NPs in two samples as a function of temperature during the in situ heating experiment. C) Schematic illustration showing the coarsening pathway of Pt NPs loaded on ceria through migration and diffusive fusion. D) HAADF‐STEM images at a high magnification showing coarsening of Pt NPs in the surface‐loaded sample.

In order to better understand the mechanistic pathways for the enhancement of thermal stability in the surface‐embedded sample, in situ heating experiment was performed and monitored by aberration‐corrected STEM at different temperatures. The sample was fast ramped up to a given temperature varying from 150 to 600 °C, and kept thermostatic for 10 min to equilibrate before electron beam was turned on for image recording. The morphological evolution of both the surface‐embedded and loaded samples at elevated temperatures were monitored using bright field (BF) STEM with a lattice resolution, as presented in **Figure** **4**A. The surface‐embedded sample shows no significant change before around 500 °C. The average sizes of Pt NPs on the surface‐loaded sample, however, grow constantly starting even at low temperature (e.g., 200 °C). To better show these trends, we tracked the size evolution of Pt NPs statistically and summarized the results as in Figure 4B. It is worthy to first point out that the size distribution of the Pt NPs achieved from the in situ experiment shows a good accordance with that from ex situ heating to 500 °C for both samples. Average size of the Pt NPs on the surface‐loaded sample shows a quasilinear increase as a function of temperature, which suggests that the coarsening is presumably dominated by the same mechanisms throughout the measured temperature range. The surface‐embedded sample, on the contrary, showed significantly lower slope before a rough turning point between 450 and 500 °C (region I). In addition, the standard deviation (shown as the error bars) remained below 0.3 nm as well. After the turning point (region II), higher coarsening rate with respect to the temperature was observed with increased standard deviations. Note that the size of Pt NPs in the embedded sample is less than 3 nm even at 600 °C, which is still smaller than most of the previously reported catalysts before any heat treatment (see Table S1 in the Supporting Information).

In general, two coarsening mechanisms, i.e., migration–coalescence and Ostwald ripening, have been observed in the sintering of supported noble‐metal NPs.^39^, ^40^, ^41^, ^42^ The previous reports suggested that coalescence and Ostwald ripening are both enabled for small particles with diameters and spacing within few nanometers, while sintering is only mediated by Ostwald ripening after interparticle spacing or anchoring of the larger NPs precludes coalescence.^43^, ^44^ The migration of NPs was also suggested to be mediated by chemical potential‐driven surface diffusion of atoms,^44^ leading to the reduction in quantity of the particles and the increase in their average size, as illustrated in Figure 4C. Consistently, spatial movement and rearrangement of Pt NPs on the surface‐loaded sample was observed when their average size was lower than 2–3 nm. As shown in Figure 4D, particle 2 was initially separated from particle 1 at 150 °C. When increasing the temperature, particle 2 moved toward particle 1 as indicated by orange arrows, and finally they joint to form a single large particle (orange dashed oval). Multiple Pt NPs can also undergo stepwise coalescence. At 150 °C, a cluster of NPs first bound with particle 4, as shown by green arrows. At higher temperature, the distance between particle 3 and enlarged particle 4 decreased and eventually a combined particle was observed at 450 °C. In the surface‐embedded sample, the relative position of Pt NPs remained nearly fixed relative to the loaded sample, and the size of these NPs increased very slowly before the temperature reached about 500 °C. This observation suggests that coarsening between Pt NPs through migration and coalescence is significantly suppressed at relatively low temperatures in the embedded sample. Only at higher temperatures exceeding 500 °C, Pt NPs were enlarged at a higher rate and their positions changed significantly. This stronger coarsening among Pt NPs may be related to the motion and coarsening among CeO_2_ grains (Figure S19, Supporting Information), since the embedded Pt NPs are greatly influenced by the structure of the surrounding CeO_2_. The polycrystalline structure of the CeO_2_ nanorods causes such variation at elevated temperatures, which has the potential to be addressed by further optimization of structural design and synthesis. As such, there is still room for further improved thermal stability of such nanostructures. Despite of this, our in situ experimental results have strongly suggested that embedding ultrafine Pt NPs at CeO_2_ surface can push up the stable temperature limit by at least 300 °C.

## 3 Conclusion

In summary, we have demonstrated the synthesis of ultrafine and high content Pt NPs partially embedded at the surface of CeO_2_ nanorods through an S–S interfacial redox reaction. Such embedded nanostructures showed much higher thermal stability against the sintering process relative to the conventional ones with ultrafine Pt NPs loaded on CeO_2_, thereby exhibiting the significantly enhanced properties toward hydrogenation of *p*‐nitrophenol after heat treatment. The key to endow the embedded nanostructures with high thermal stability in a broad temperature range is the suppression of particle migration and coalescence due to the unique architecture, which was clearly revealed by in situ heating experiments with electron microscopy. This work offers new insights to the design and understanding of sinter‐resistant catalysts based on metal NPs embedded at oxide surfaces. One may further apply this synthesis strategy to other materials systems involving similar valence‐changing oxide supports and noble‐metal NPs,^25^, ^26^, ^27^, ^45^, ^46^ depending on the specific target reactions. Taken together, this report will lead to an enhanced understanding of both the design rules and the mechanistic pathways for sinter‐resistant hybrid nanocatalysts.

## 4 Experimental Section

*Chemicals*: Cerium nitrate (Ce(NO_3_)_3_·6H_2_O, 99.95% metals basis), cetyl trimethylammonium bromide (CTAB, 99%), and sodium borohydride (NaBH_4_, 98%) were supplied by Aladdin Chemicals. Potassium tetrachloroplatinate (K_2_PtCl_4_, 99.99% trace metals basis) was supplied by Aldrich. PVP (*M*_W_ ≈ 40 000) was supplied by Sigma‐Aldrich. Sodium hydroxide (NaOH, analytical reagent) was supplied by Sinopharm Chemical Reagent. These chemicals were used without further purification. Ultrapure water was produced by a Millipore Synergy water purification system with a resistivity of 18.2 MΩ cm (25 °C).

*Synthesis of Surface‐Embedded Pt/CeO_2_ Hybrid Nanostructures*: In a typical synthesis, 170 mg of CTAB was dissolved in 14 mL of water, followed by the addition of 1 mL of 0.01 m Ce(NO_3_)_3_ aqueous solution. The mixture was heated to 70 °C under Ar protection for 20 min under magnetic stirring. After that, 1 mL of 0.1 m NaOH aqueous solution was pumped at a rate of 1 mL h^−1^. The whole pumping process took 1 h. In the half way of pumping (30 min), 1 mL of 0.01 m K_2_PtCl_4_ aqueous solution was injected using a pipette. After the end of pumping, the mixture was kept at 70 °C for 1 h and then cooled down to room temperature under Ar protection. The product was obtained by centrifugation and was washed several times with ethanol for further use.

*Synthesis of CeO_2_ Intermediate Nanorods*: Large‐scale synthesis of *CeO*_2_ intermediate nanorods was performed as follows. 180 mg of CTAB was dissolved in 5 mL of water, followed by the addition of 5 mL of 0.05 m Ce(NO_3_)_3_ aqueous solution. The mixture was heated to 70 °C under Ar protection for 20 min under magnetic stirring. 5 mL of 0.1 m NaOH aqueous solution was pumped at the rate of 1 mL h^−1^. The mixture was then cooled down to room temperature under Ar protection. The product was obtained by centrifugation and was washed several times with ethanol for further use.

*Synthesis of Surface‐Loaded Pt/CeO_2_ Hybrid Nanostructures*: In a typical synthesis, 10 mL of ceria intermediate nanorod aqueous suspension was stirred at 95 °C for 30 min. After adding 1 mL of 0.01 m K_2_PtCl_4_ aqueous solution, the reaction was kept at 95 °C for another 1 h without the addition of any other reducing agent. The mixture was cooled down to room temperature and washed three times with ethanol by centrifugation for further use.

*Synthesis of Encapsulated Pt/CeO_2_ Hybrid Nanostructures*: The typical synthetic procedure is similar to that of surface‐embedded structure, except that the 1 mL of 0.1 m NaOH aqueous solution was premixed with 1 mL of 0.01 m K_2_PtCl_4_ aqueous solution as the pumped solution. No further K_2_PtCl_4_ was added.

*Ex Situ Heat Treatment*: Samples in a powder form collected from centrifugation were heated at the rate of 8.3 °C h^−1^. Then, they were kept at 500 °C for 3 h before cooling to room temperature. The whole process was performed under Ar protection.

*Characterization*: TEM was performed by a Hitachi HT7700 operated under 100 kV. HRTEM and SAED were taken by a JEOL JEM‐2100F operated under 200 kV. HAADF‐STEM was performed by either an FEI Titan G^2^ with spherical aberration (*C*_s_) correctors and a super‐X EDX detector operated under 200 kV, or an FEI Tecnai G^2^ F20 with an EDX detector operated under 200 kV. The Pt loading in the samples were determined using inductively coupled plasma mass spectroscopy (ICP‐MS, Thermo X Series). XPS was taken by a ThermoFisher Escalab 250Xi. Powder XRD was performed on a Rigaku D/max X‐ray diffractometer with graphite monochromatized Cu *K*_α_ radiation (λ = 1.54178 Å). UV–vis spectra were obtained by either a Hitachi U‐4100 or an Ocean Optics DH‐2000 spectrometer. FTIR was done by a Bruker IFS 66V/S.

*Ex Situ CO Adsorption Experiment*: Three types of Pt/CeO_2_ hybrid structures including the surface‐embedded, surface‐loaded, and encapsulated were dispersed into ethanol, respectively. The dispersions were bubbled with constant CO gas flows for 2 h. Then, the dispersions were drop casted on a KBr substrate and dried in a CO atmosphere. After drying, all samples were sealed in a box before FTIR measurements were performed. All IR absorption spectra were normalized before comparison.

*Catalytic Study*: The catalytic study follows the established procedures with slight modifications.[[qv: 5a,19]] Freshly prepared aqueous solution of *p*‐nitrophenol (0.01 m, 20 µL) and NaBH_4_ (0.2 m, 20 µL) were mixed together in 3 mL of water in a cuvette. An aqueous catalyst suspension with predetermined Pt atomic concentration by ICP‐MS was sonicated and stirred to ensure catalyst dispersity. A volume of this suspension containing 9.754 µg of Pt was quickly added to the cuvette. The reaction was monitored in situ by absorption peak intensity at 400 nm in a UV–vis spectrometer. The whole process was performed at 25 °C. The amount of NaBH_4_ was in excess to keep the reaction as first order. Previous reports have suggested that the apparent first‐order rate constant (*k*_app_) is proportional to the concentration of Pt, thus the Pt‐concentration‐specific rate constant *k*_1_ was defined to take the exact concentration of catalyst into consideration as in Equation (2)[[qv: 5a]]

(2) $$k_{1}\;=\;k_{\mathrm{app}}/M$$

where *M* is the atomic concentration of Pt in g L^−1^ in the whole reaction solution and the apparent rate constant *k*_app_ follows Equation (3)

(3) $$-dc\left(t\right)\mathrm{/d}t\;=\;k_{\mathrm{app}}c\left(t\right)$$

The conversion efficiency was calculated by comparing the initial characteristic intensity at 400 nm with the one at 600 s.

*In Situ Heating Experiment*: In situ heating experiment under STEM was performed by an FEI Titan G^2^ with spherical aberration (*C*_s_) correctors with a heating holder (DENSsolutions). In the absence of electron radiation, the sample was fast ramped up and kept at an aimed temperature for 10 min to equilibrate. The electron beam was then illuminated on the sample before the scanning images were captured.

## Conflict of Interest

The authors declare no conflict of interest.

## Supporting information

Supplementary
