# Supplementary material for: Embedding Ultrafine and High‐Content Pt Nanoparticles at Ceria Surface for Enhanced Thermal Stability
Source: Adv Sci (Weinh). 2017 May 4;4(9):1700056. doi: 10.1002/advs.201700056 (PMC5604392; doi:10.1002/advs.201700056)
Supplement: Supplementary file 1 — Supplementary [file ADVS-4-na-s001.pdf]

Copyright WILEY-VCH Verlag GmbH & Co. KGaA, 69469 Weinheim, Germany, 2017.

ADVANCED SCIENCE
Open Access

Supporting Information

for *Adv. Sci.,* DOI: 10.1002/advs.201700056

Embedding Ultrafine and High-Content Pt Nanoparticles at Ceria Surface for Enhanced Thermal Stability

Jingshan S. Du, Ting Bian, Junjie Yu, Yingying Jiang, Xiaowei Wang, Yucong Yan, Yi Jiang, Chuanhong Jin, Hui Zhang,* and Deren Yang*

Copyright WILEY-VCH Verlag GmbH & Co. KGaA, 69469 Weinheim, Germany, 2017.

Supporting Information

Embedding Ultrafine and High-Content Pt Nanoparticles at Ceria Surface for Enhanced Thermal Stability

Jingshan S. Du, Ting Bian, Junjie Yu, Yingying Jiang, Xiaowei Wang, Yucong Yan, Yi Jiang, Chuanhong Jin, Hui Zhang*, Deren Yang*

This file contains:

Figure S1 to S19

Table S1 to Table S3

References

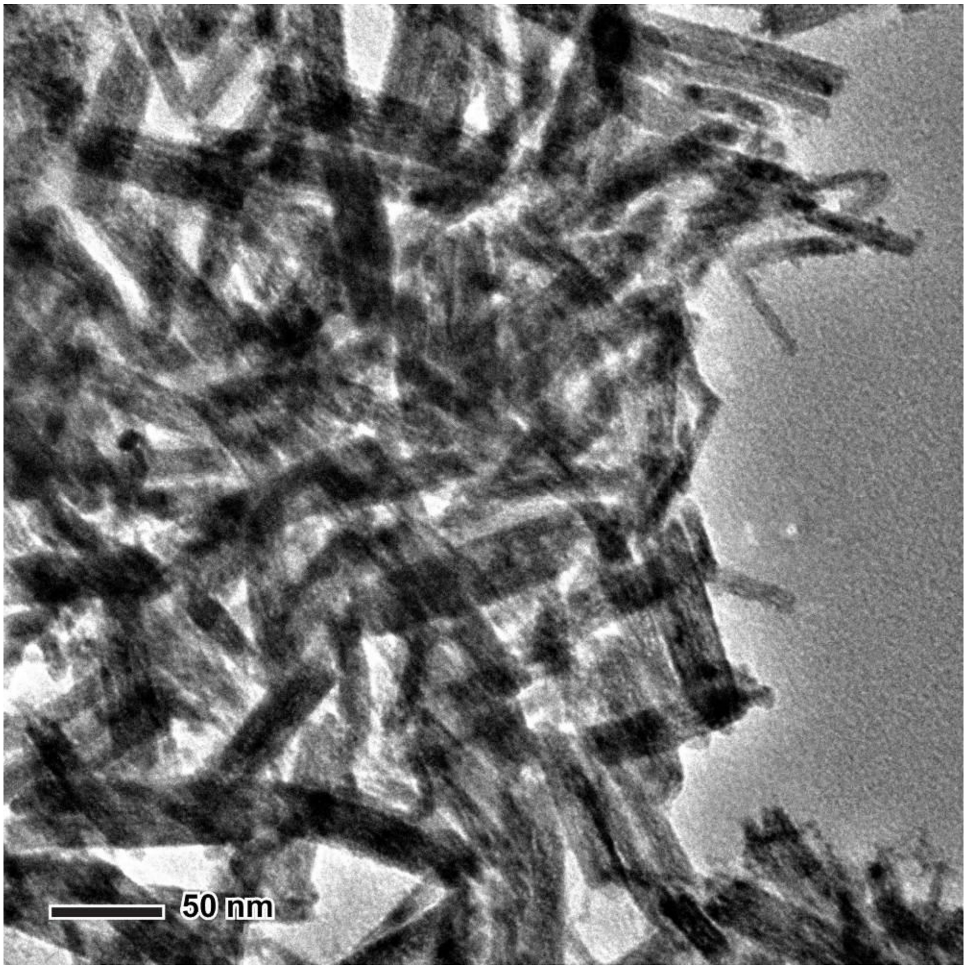

Figure S1. Low-magnification TEM image of the surface-embedded Pt/CeO_2 nanorods.

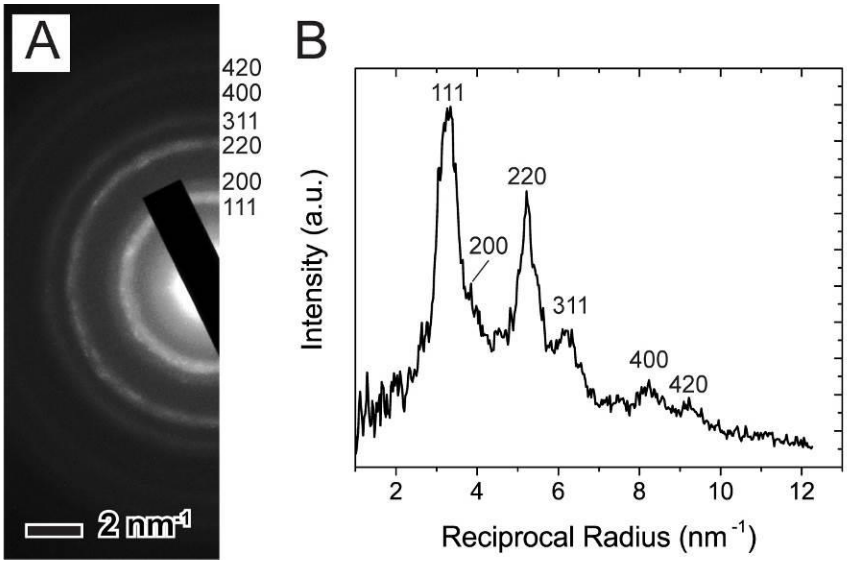

Figure S2. (A) SAED pattern of an area covering a large amount of surface-embedded Pt/CeO_2 nanorods and (B) a corresponding line profile (baseline subtracted assuming exponential decay). Fluorite-type CeO_2 reflections are marked besides each ring and in the profile.

Table S1. Comparison of some key parameters such as size and loading of metal nanoparticles and stable temperature for surface-embedded Pt/CeO_2 nanostructures in this work with the typical supported catalysts in previous reports. The reference number here refers to the sequence of the additional references in SI.

**Catalysts**	**Metal NP size**	**Metal NP loading**	**Stable temperature**	**Ref. in SI**
Surface-embedded Pt NPs on CeO_2 nanorods	1-2 nm	14 wt%	450-500 °C	This work
Porous SiO_2-coated Pt NPs on TiO_2 nanofibers	3.1 nm	1.3 wt% (not incl. SiO_2)	> 750 °C	1
		3.6 wt% (not incl. SiO_2)	~ 550 °C	
Pre-calcinated Pt NPs on CeO_2 nanocubes	1-2 nm	27 wt%	~ 450 °C	2
Pd NPs @ hollow CeO_2 shells	4.9 nm	0.4 wt%	> 550 °C	3
Pt NPs in hollow CeO_2 microfibers	3.5 nm	0.98 wt%	~ 700 °C	4
Pt NPs on TiO_2 with partial SiO_2 coverage	3.4 nm	N. A.	~700 °C	5
Multi-Pt NPs @ CeO_2 shells	3-5 nm	8.7 wt%	~ 600 °C	6

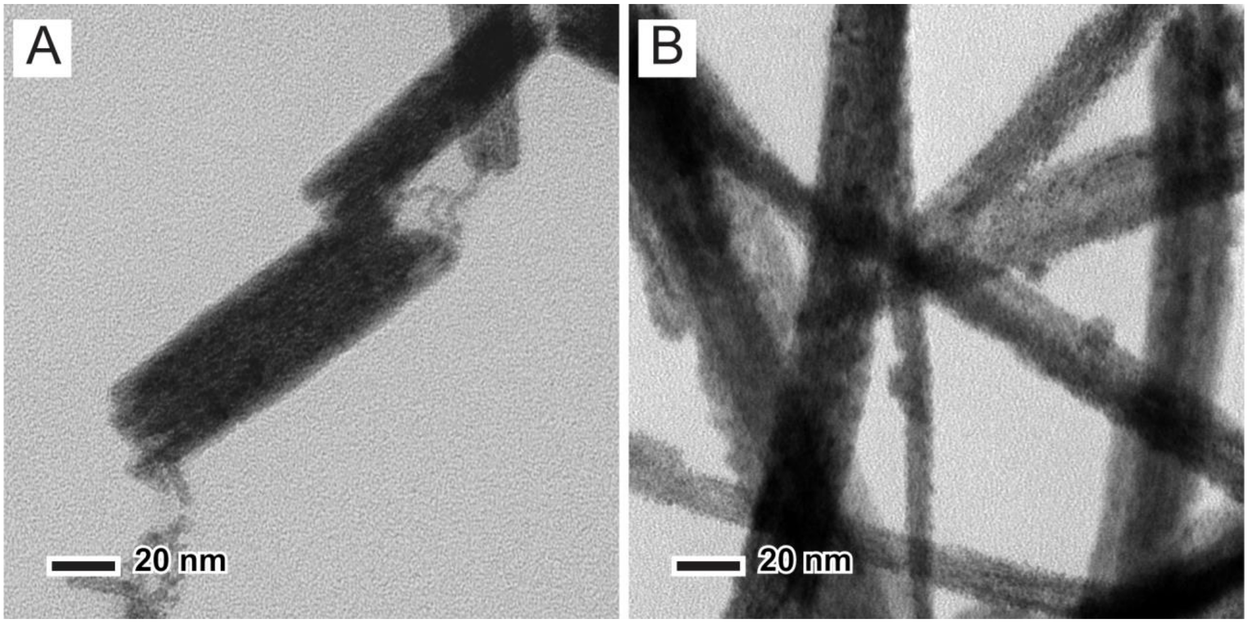

Figure S3. TEM images of (A) surface-embedded and (B) surface-loaded Pt/CeO_2 hybrid nanostructures.

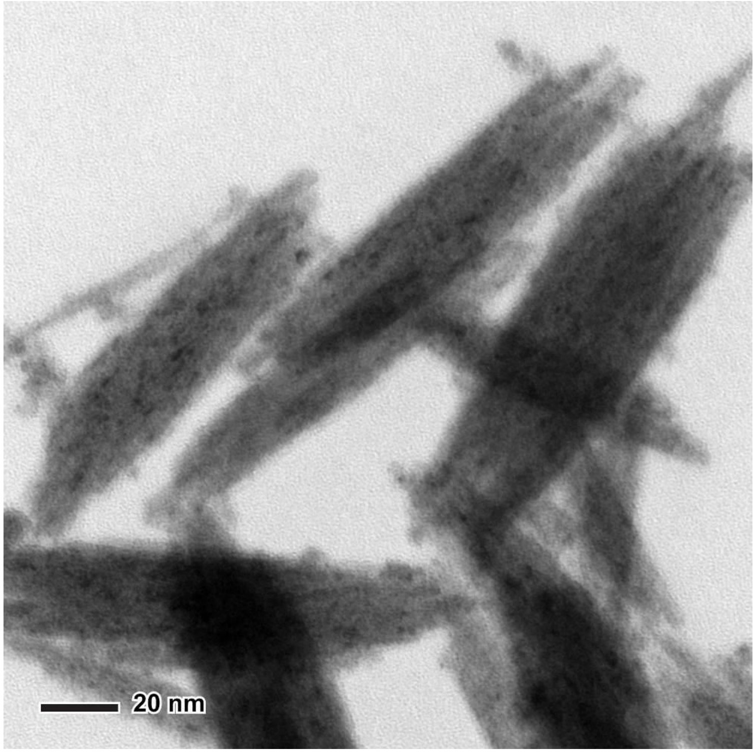

Figure S4. TEM image of the encapsulated Pt/CeO_2 hybrid nanostructures.

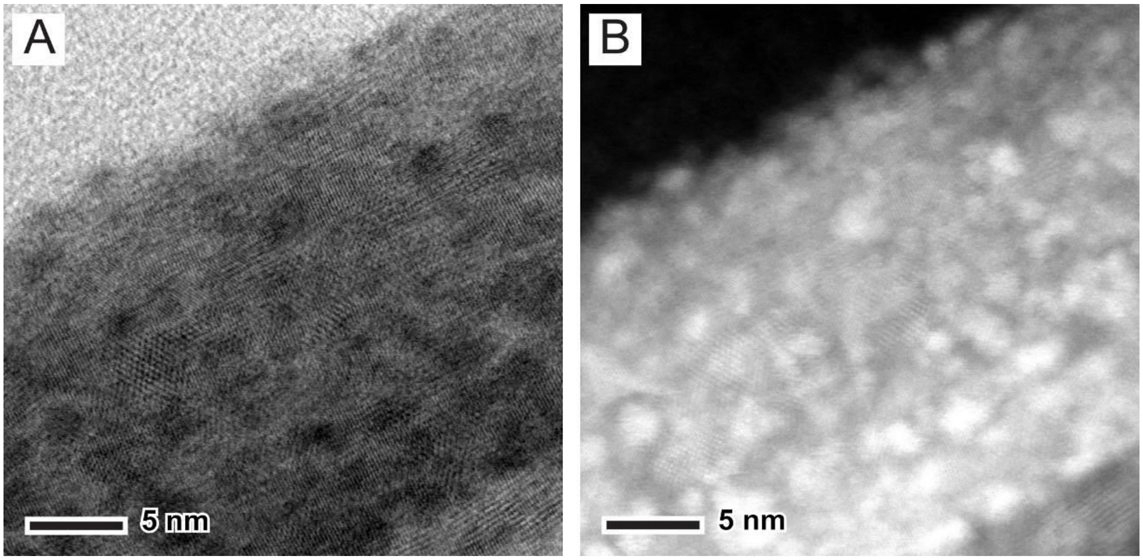

Figure S5. (A) BF- and (B) HAADF-STEM images showing a typical local area on the surface-embedded Pt/CeO_2 hybrid nanostructure.

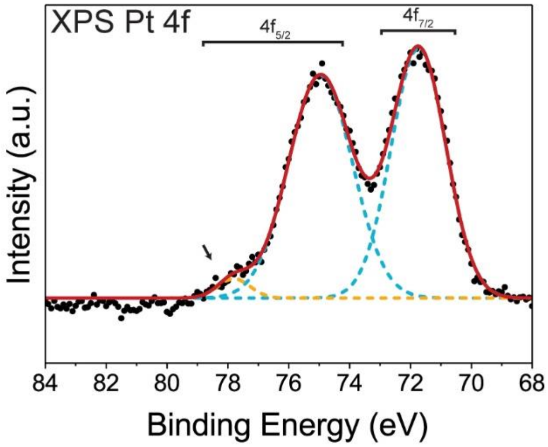

Figure S6. XPS analysis of the Pt 4f core level in the encapsulated Pt/CeO_2 hybrid nanorods. Black dots represent measured data and red curves are the fitting results. Dashed curves with different colors refer to the resolved peaks. A high-energy component similar to that in the surface-embedded sample is indicated with a black arrow.

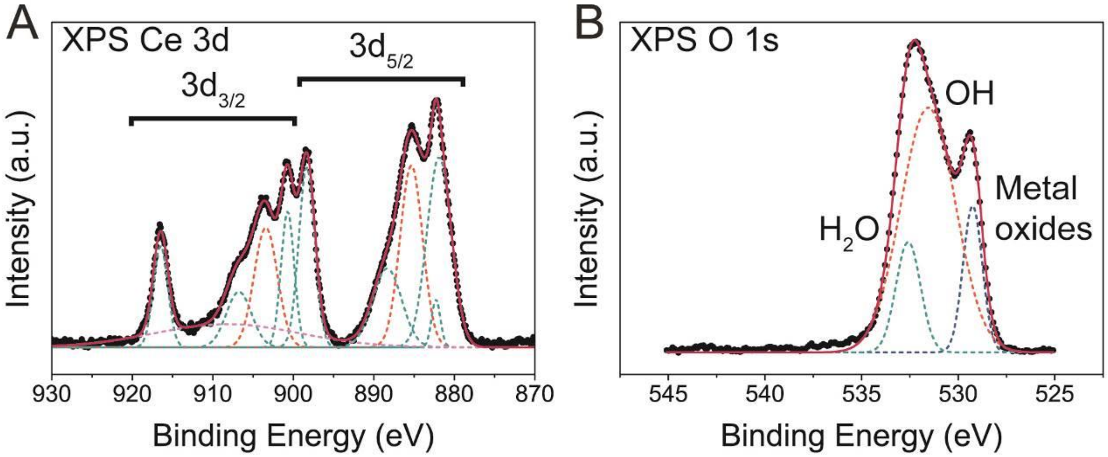

Figure S7. XPS analysis of (A) Ce 3d and (B) O 1s core levels in the surface-embedded Pt/CeO_2 hybrid nanostructures. Black dots represent measured data and red curves are the fitting results. Dashed curves with different colors refer to the resolved peaks. The very broad component in pink dashes in (A) is likely originated from the instrumental background.

XPS analysis. The XPS spectra were calibrated by aligning the C 1s peak at 284.8 eV. In Figure S7A, each resolved peak in the two spin states is assigned to either Ce^{III} or Ce^{IV}, according to previous experimental data.[7-9] A very broad component (pink dashes) is present, which is likely originated from the instrumental background. The existence of both valences of Ce is expected from natural ceria samples and their hybrids with Pt due to the formation of oxygen vacancies.[7-9] In Figure S7B, the three resolved peaks in O 1s spectra are ascribed to the bonding in H_2O, hydroxyl groups and metal oxides, respectively.[10] The peak at 531.55 eV revealed the rich existence of hydroxyl groups on the hybrid nanorods. It is also indicated that water molecules are absorbed on the surface due to the smaller peak at 532.58 eV. These results are consistent with the infrared spectroscopy results (see Figure S18). In the main text Figure 1E, two dominant peaks consisting of Pt^0 (blue dashes) and Pt^{II} (green dashes) peaks are resolved from both samples according to their binding energy.[11] Overall shift was noticed compared to pure Pt or its oxides, which is expected from the strong interaction effect with ceria. The existence of a highly oxidized Pt state, most likely Pt^{IV} (marked by an arrow) suggests that considerable embedding of Pt by the surrounding ceria is formed, since such split peaks have not been observed from the loaded sample in our experiment or other previous literature reports.[12] It has been found that Pt

can partially disperse into the lattice of ceria,[13] therefore the interface of oxide and Pt metal should reasonably have multiple Pt-O bonds per Pt atom depending on local coordination environment, thus leading to an energy shift of Pt $4f_{5/2}$ core level. Tiny shift of $4f_{7/2}$ due to oxidation has also been observed.

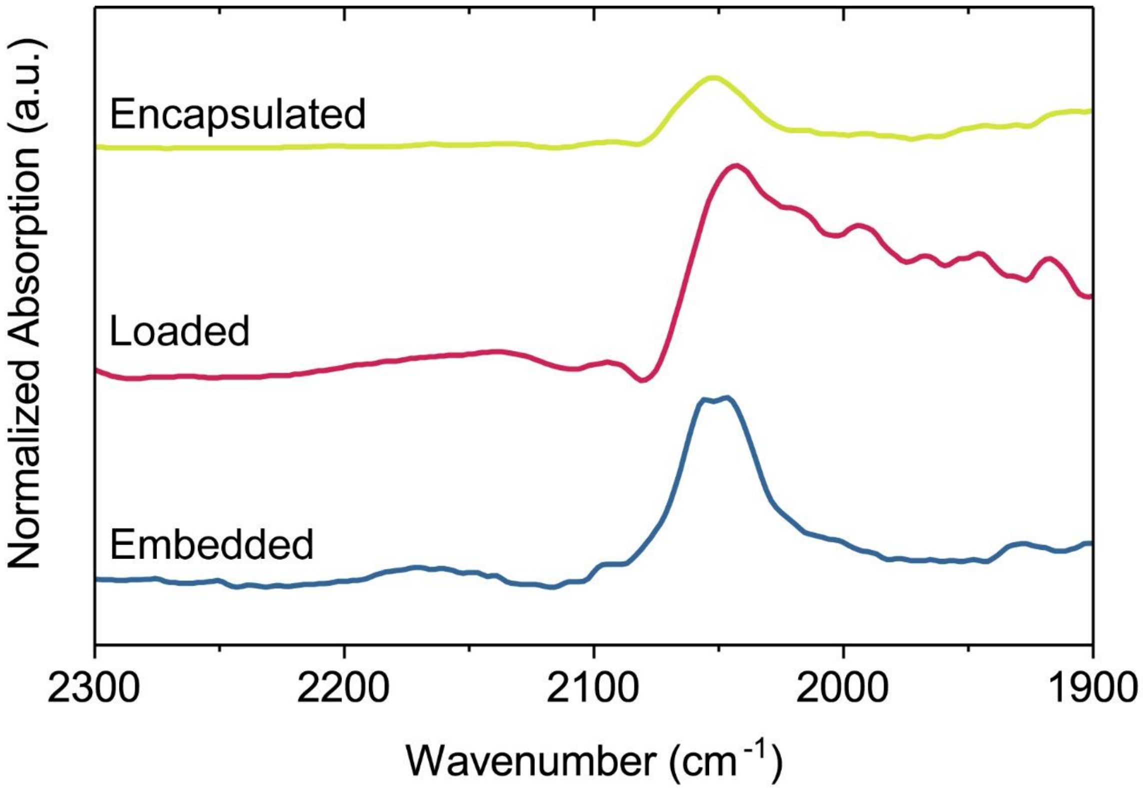

Figure S8. FTIR spectra of the surface-embedded, surface-loaded, and encapsulated samples after *ex-situ* CO adsorption treatment.

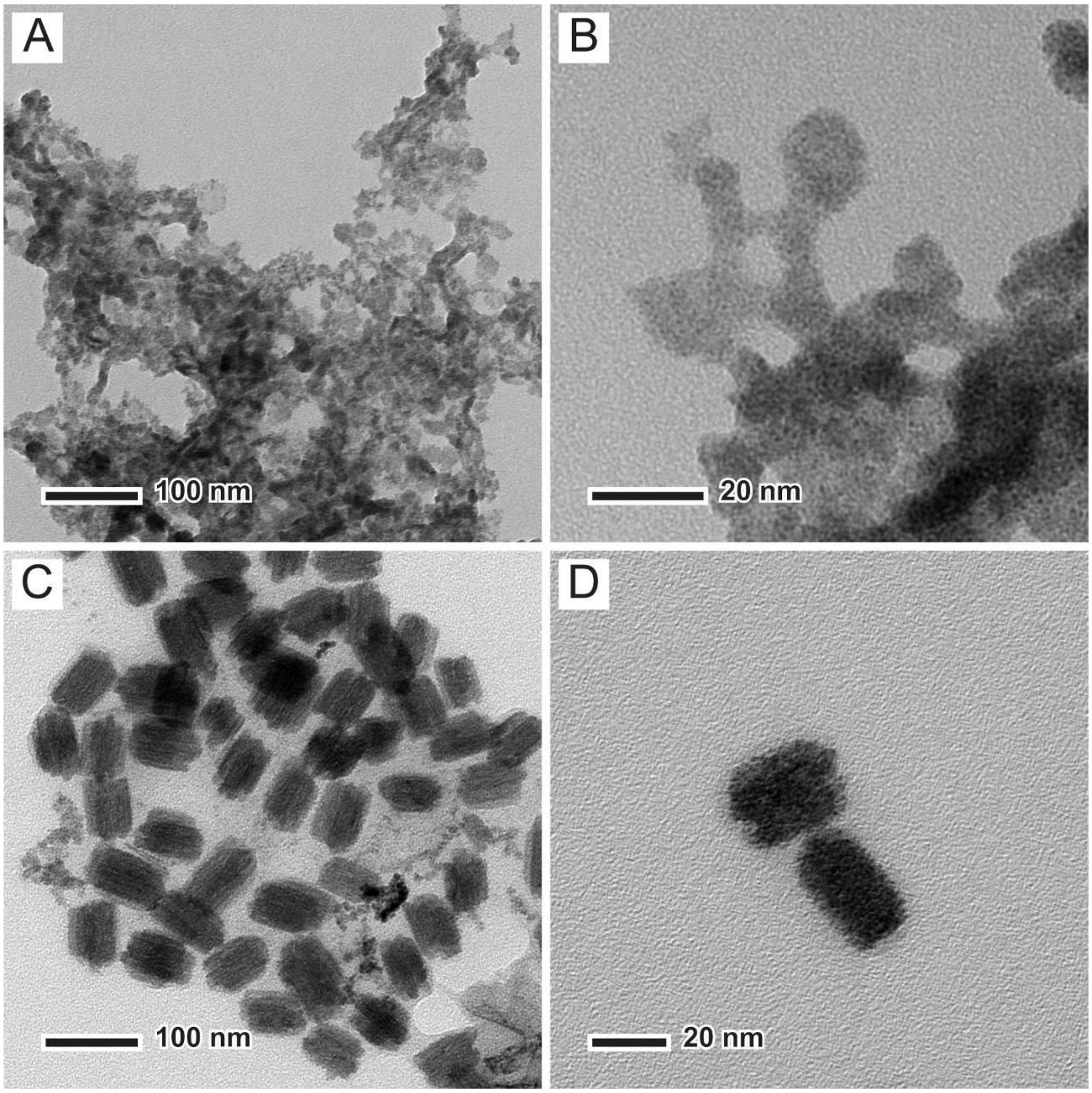

Figure S9. (A, B) TEM images of the network-like Pt/CeO_2 nanostructures formed by replacing CTAB with AHA. (C, D) TEM images of Pt/CeO_2 nanoparticles formed by replacing CTAB with PVP.

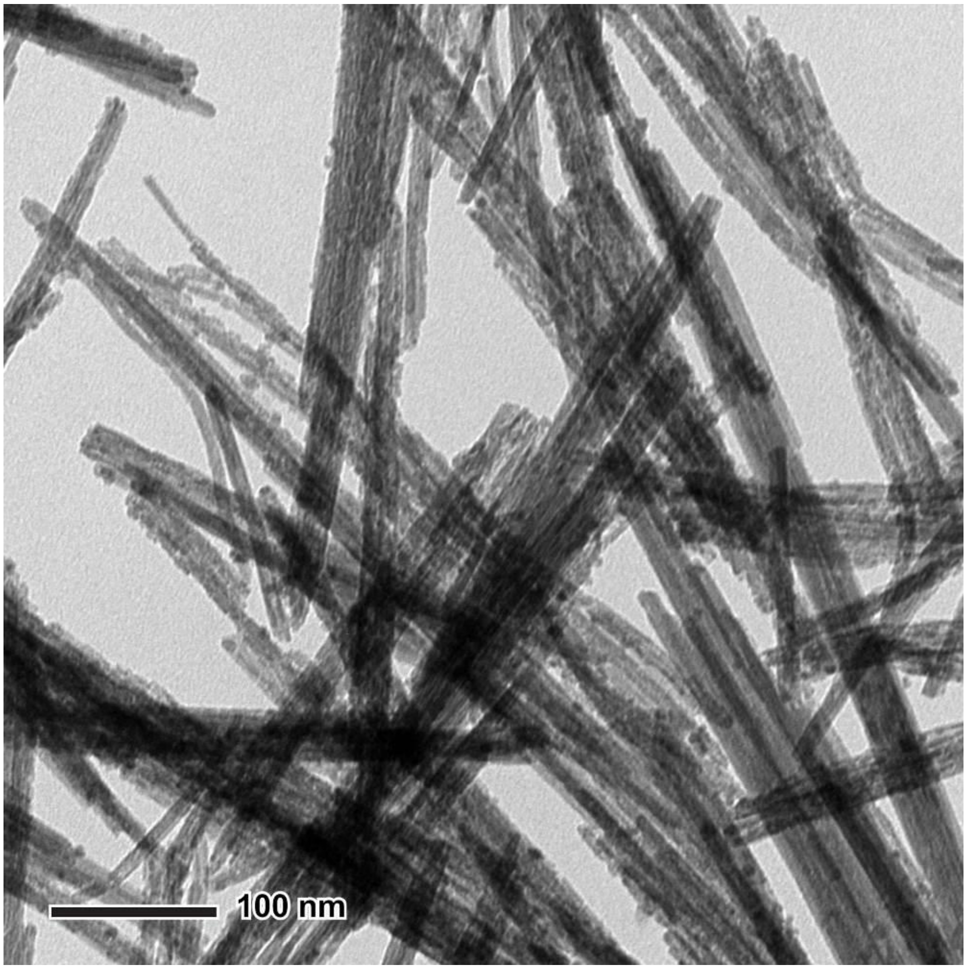

Figure S10. TEM image of ceria intermediate nanorods in the absence of Pt precursor.

Large-scale synthesis of ceria intermediate nanorods. Pure ceria intermediate nanorods were synthesized by a large-scale approach in the absence of Pt precursor as shown in Figure S10. The average diameter of these nanorods is similar to that of the embedded Pt/CeO_2, whereas the length is much longer. It suggests that higher concentration of Ce^{3+} ions in the large-scale synthesis than in standard procedure for the embedded Pt/CeO_2 leads to the elongation of ceria nanorods. This method is of high yield, and the production capability is *ca.* 45 mg per run in a 25-mL flask. The product can be collected by low-rate centrifugation or precipitation with ethanol and can also be easily re-dispersed in water.

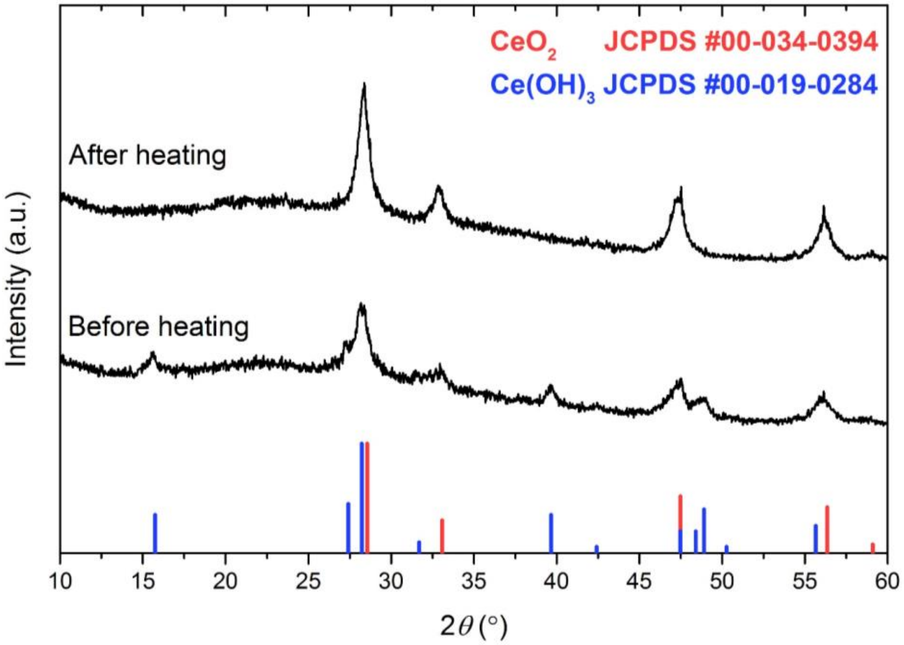

Figure S11. XRD patterns of the ceria intermediate nanorods before and after heat treatment at 500 °C. Standard XRD patterns of CeO_2 (JCPDS #00-034-0394) and $Ce(OH)_3$ (JCPDS #00-019-0284) are also given for reference.

XRD analysis. XRD patterns of both the ceria intermediate nanorods before and after heat treatment at 500 °C were recorded as in Figure S11. By referencing the standard diffraction data (shown as the bars at the bottom of the figure), the as-prepared nanorods show both reflections associated with CeO_2 and $Ce(OH)_3$ phases. This result indicates the abundant existence of hydroxyl groups and Ce^{III}, which is the active species contributing to the reduction and attachment of Pt NPs.[6] Heat treatment at 500 °C is able to convert all hydroxide into CeO_2 phase. In addition, CeO_2 reflections appear sharper, which indicates larger grain size and improved crystallinity.

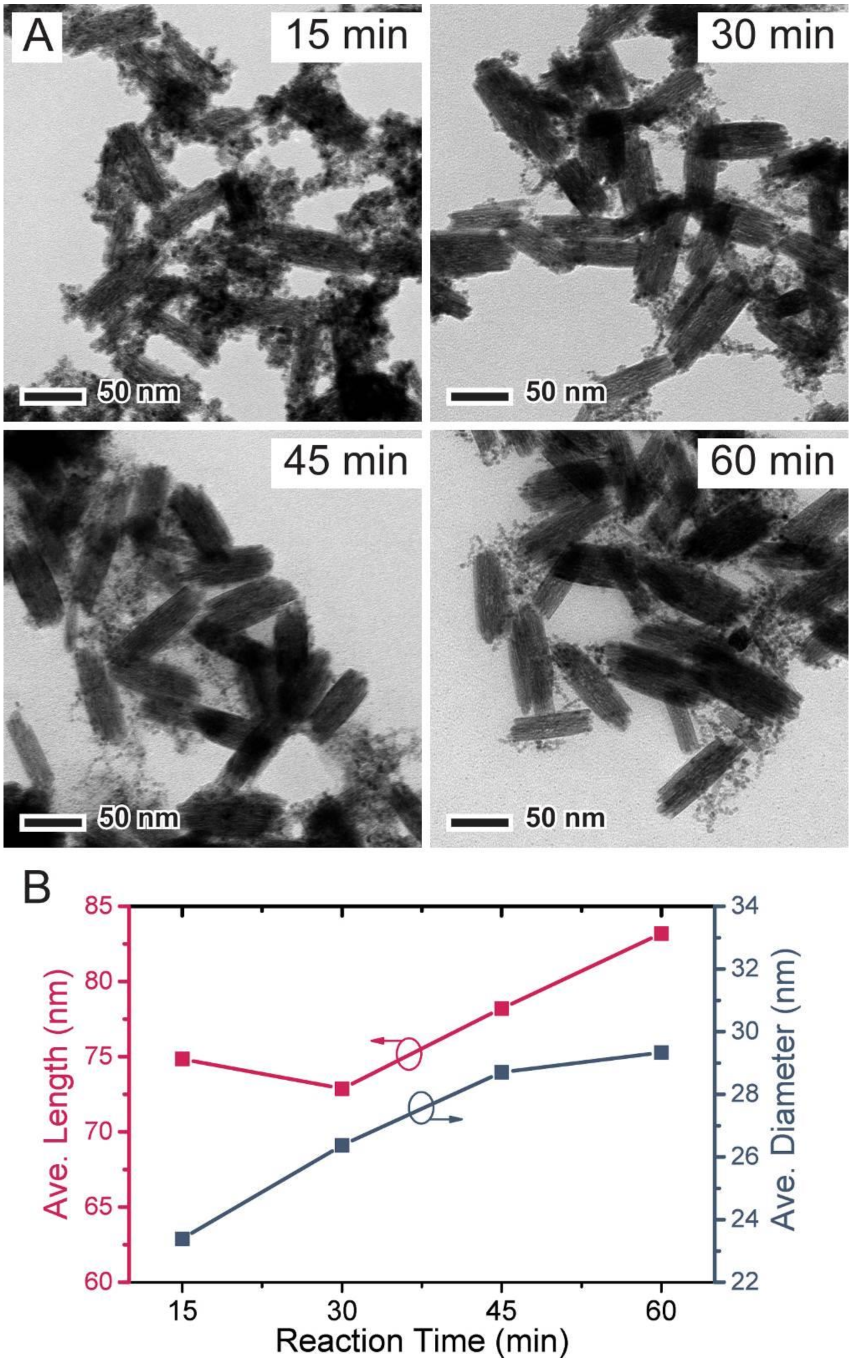

Figure S12. Formation of the ceria intermediate nanorods. (A) TEM images of the ceria intermediate nanorods at different time of the reaction. (B) Average length and diameter of ceria nanorods as a function of reaction time.

Evolution of ceria intermediate nanorods. The slow injection of NaOH controls the reaction kinetics so that the size of ceria intermediate nanorods increases during the whole process of reaction, as suggested by the time-series investigation of the formation process in Figure S12. During the first half hour after NaOH injection starts, short nanorods with average diameter of ca. 75 nm are formed. The latter half hour witnesses the growth of nanorod diameter to about 83 nm. The average length of the rods is also increasing during the reaction time. This result suggests that continuous growth of ceria/cerium hydroxide still takes place after injecting Pt precursors and plays an important role in embedding and fixing the Pt NPs *in situ*. Note that there are always small NPs of ceria or cerium hydroxide formed during the reaction, which is also observed in the surface-embedded Pt/CeO_2 samples.

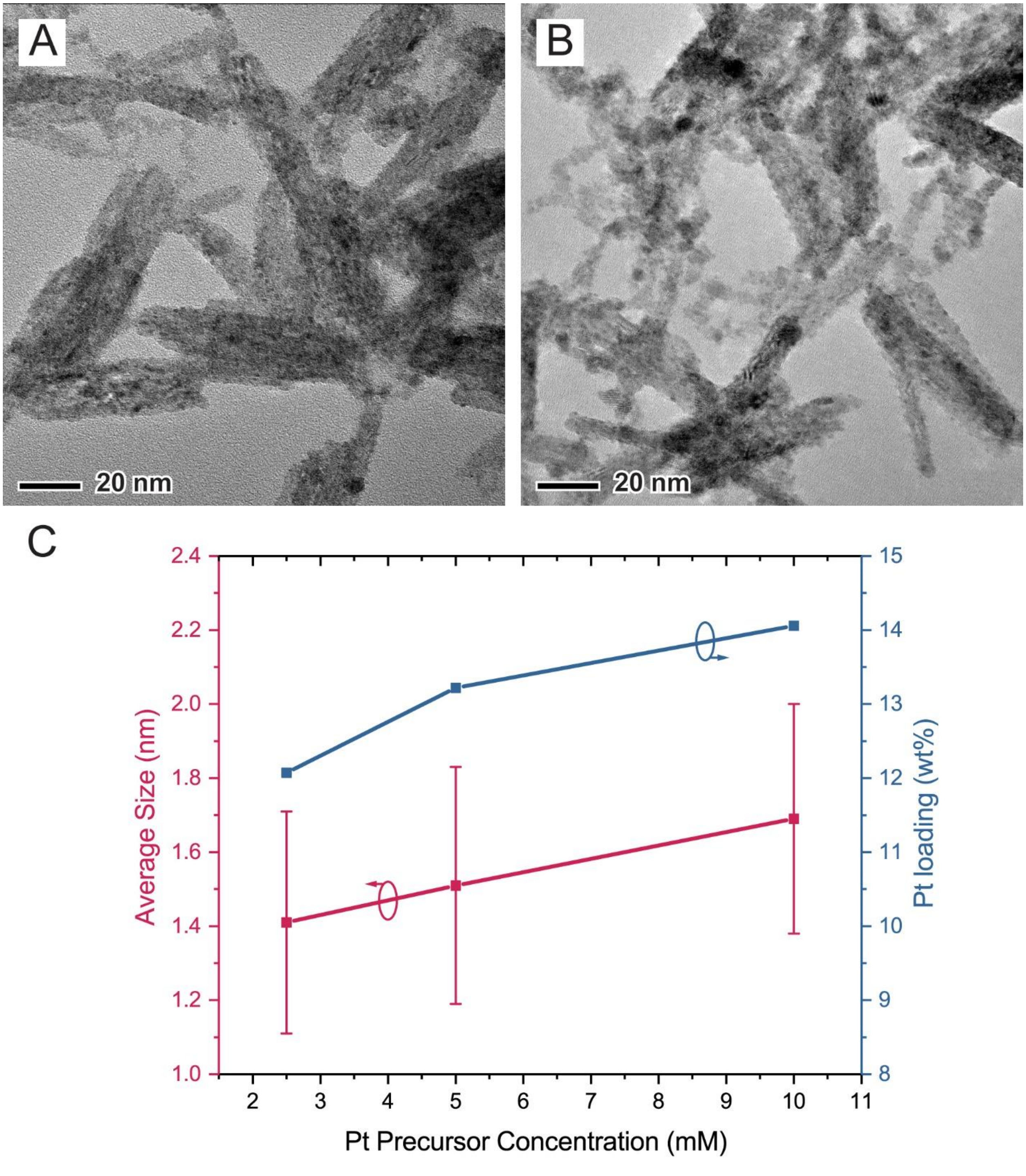

Figure S13. Tunability on Pt loading. (A, B) Typical HRTEM images showing the surface-embedded Pt/CeO_2 nanostructures with decreased Pt precursor concentration, half (5 mM, A), or a quarter (2.5 mM, B). (C) Pt Size statistics from TEM images, and Pt mass loading percentage

determined from ICP-MS as functions of the Pt precursor concentration. Error bars in size statistics represent standard deviations.

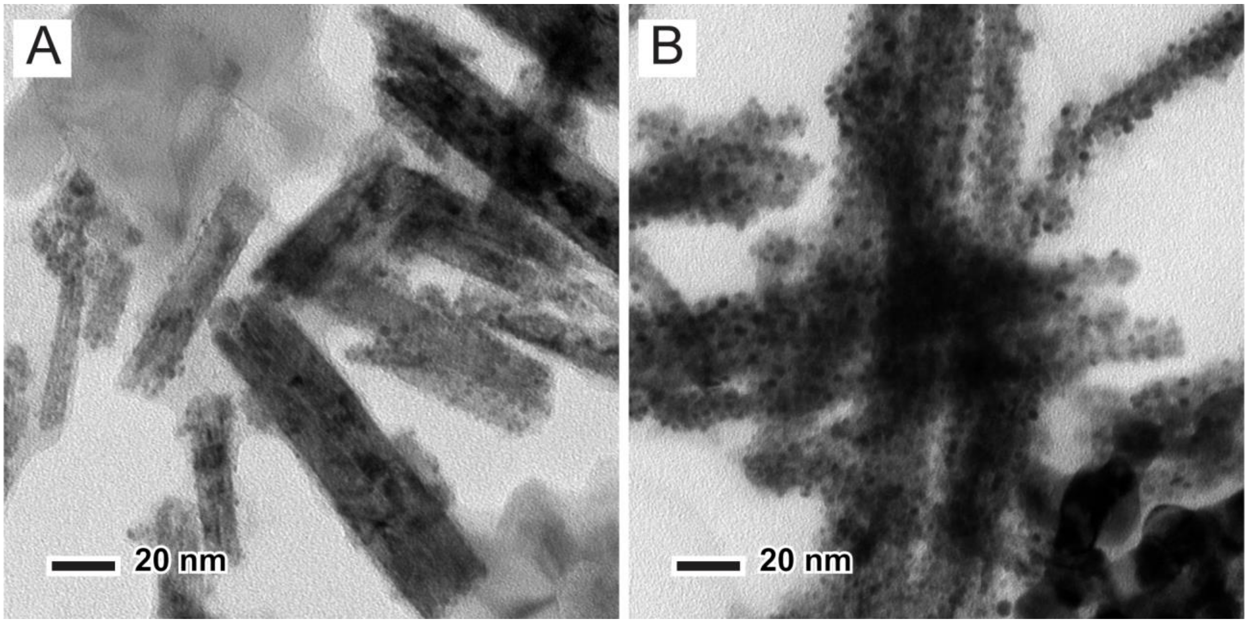

Figure S14. Typical TEM images of (A) the surface-embedded and (B) the surface-loaded sample after heat treatment at 500 °C.

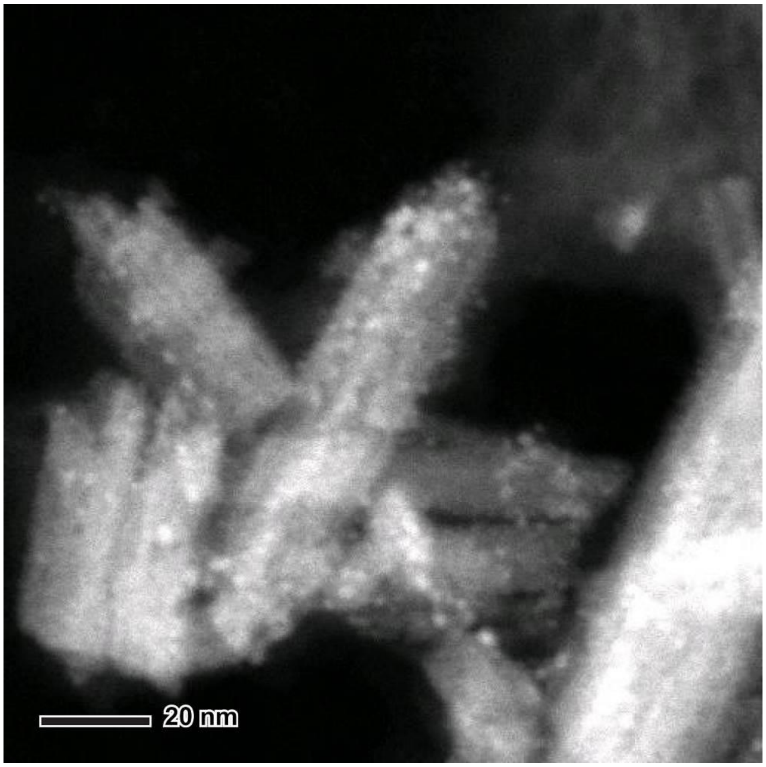

Figure S15. Typical HAADF-STEM image of surface-embedded sample after heat treatment.

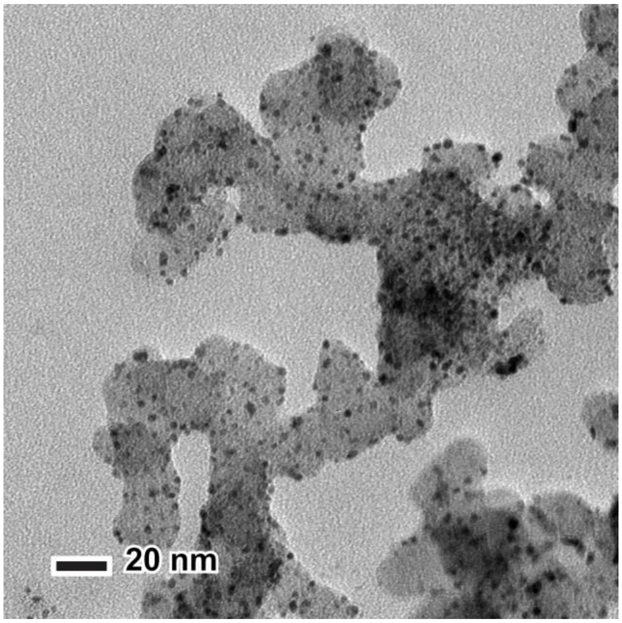

Figure S16. Typical TEM image of the commercial Pt/C catalyst tested in the catalytic experiment.

Table S2. Size statistics of Pt NPs in surface-embedded and surface-loaded Pt/CeO_2 hybrid nanorods before and after heat treatment at 500 °C. Size statistics for the commercial Pt/C catalyst is also given.

Sample	Average (nm)	Median (nm)	Standard deviation (nm)
Surface embedded	1.69	1.7	0.31
Surface embedded (heated)	2.15	2.1	0.55
Surface loaded	1.55	1.6	0.32
Surface loaded (heated)	3.28	3.2	0.69
Commercial Pt/C	2.25	2.1	0.98

Table S3. Statistics of the center-to-center distance of adjacent Pt NPs on the surface-embedded and surface-loaded Pt/CeO_2 samples, measured along the edges of the nanorods.

Sample	Average distance (nm)	Standard deviation (nm)
Surface embedded	1.80	0.36
Surface loaded	1.81	0.27

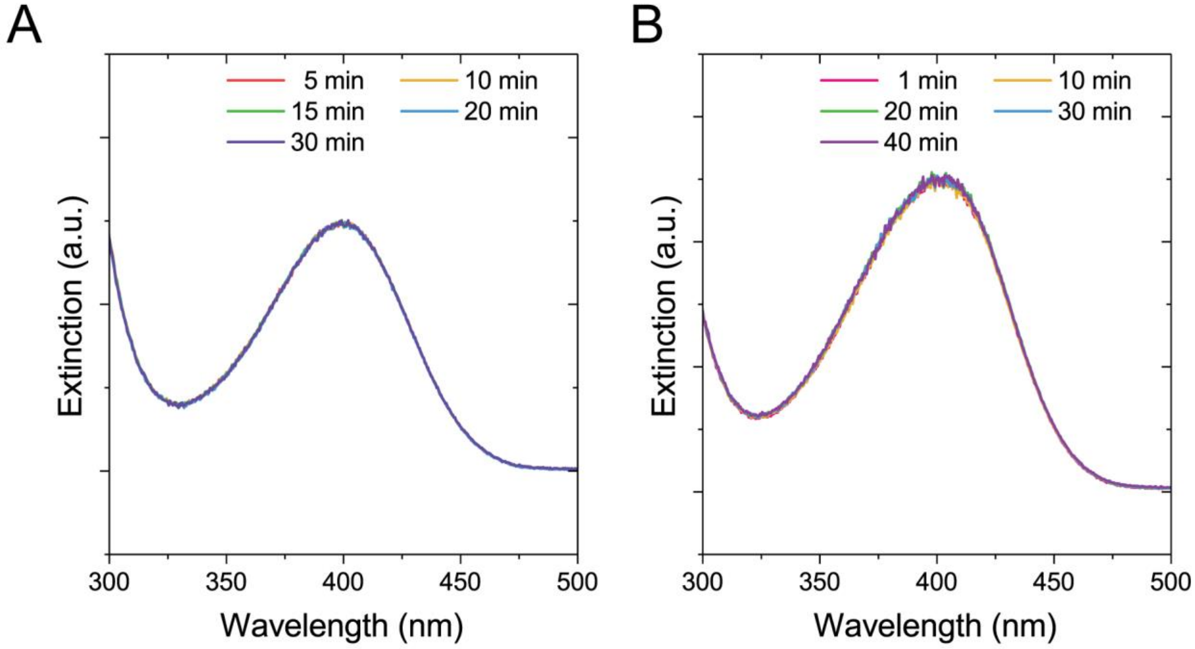

Figure S17. UV-Vis spectra achieved at different time for hydrogenation of *p*-nitrophenol in the presence of $NaBH_4$ and as-prepared CeO_2 nanorods before (A) or after (B) heat treatment at 500 °C. Both of them show no catalytic activity.

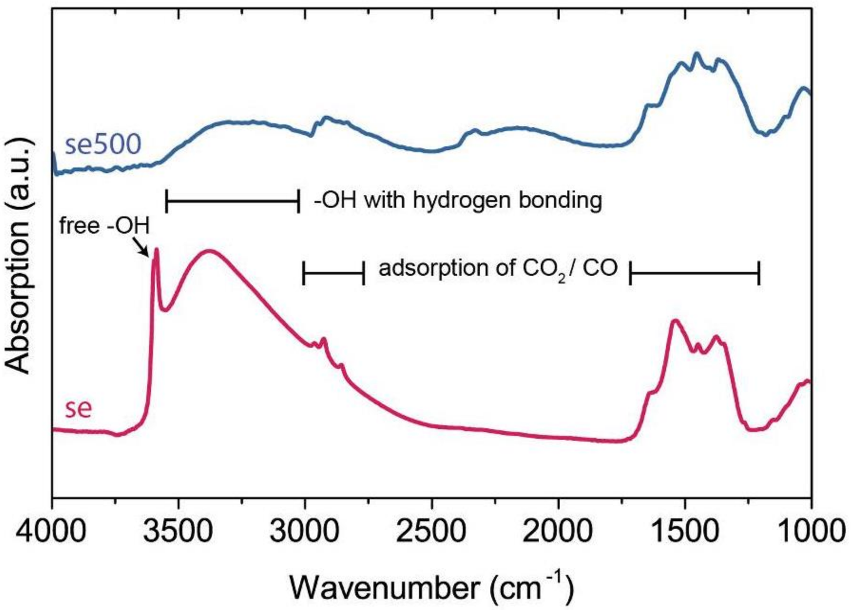

Figure S18. FTIR spectra of surface-embedded Pt/CeO_2 nanorods before (se) and after heat treatment (se500) at 500 °C.

FTIR spectroscopy analysis. Figure S18 shows FTIR spectra of the surface embedded samples before and after *ex-situ* heat treatment at 500 °C. Two samples share two groups of peaks in the range of 3000-2800 cm^{-1} and 1700-1200 cm^{-1}, respectively. These two groups of peaks can be attributed to the adsorption of carbon oxides on the surface of ceria.[14] Two samples also exhibit the vibration mode of –OH groups with hydrogen bonding as the wide peak around 3500-3000 cm^{-1}, which indicates the presence of the adsorbed water molecules from the aqueous solution or the atmosphere. The most striking change after heat treatment, however, is the disappearance of the sharp peak at *ca.* 3600 cm^{-1}, which can be attributed to free hydroxyl groups.[15,16] This result indicates the removal of hydroxyl groups from ceria after heat treatment, which might be responsible for the enhancement of catalytic activity by lowering defect density and providing more active sites and less steric hindrance on the surface.

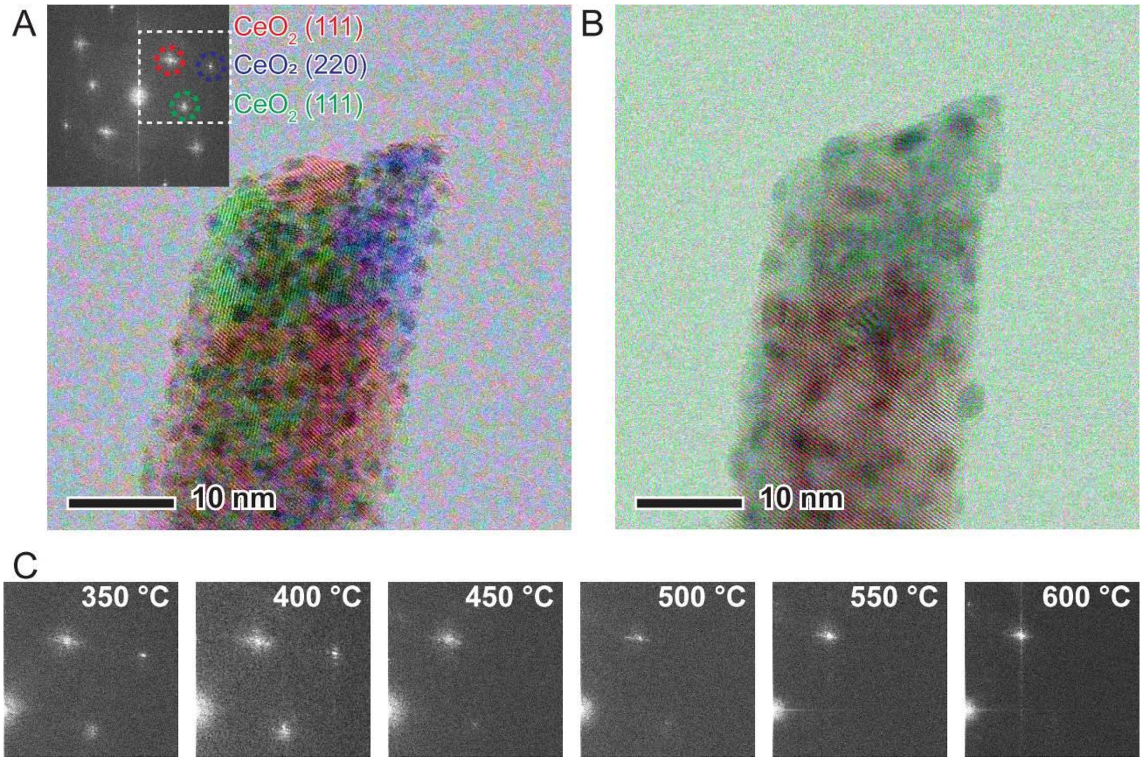

Figure S19. CeO_2 grain orientation analysis during the *in-situ* heating experiment. (A) High-resolution bright field (BF) STEM image at 400 °C showing CeO_2 grains colored by red, green and blue. Three channels are generated by filtering the FFT image around three pairs of diffractions, respectively. Inset: The corresponding FFT pattern showing the three diffractions of CeO_2 used for false color generation. Components along the middle vertical line in FFT originate from scanning artifacts and have been filtered out when presenting the STEM image. (B) High-resolution bright field (BF) STEM image at 600 °C false colored with the same diffractions in (A). Domains colored with red become dominant in most regions along the CeO_2 nanorod. (C) FFT patterns at a high magnification showing the three diffractions (white dashed box in the inset of figure A) at different temperatures during the heating experiment.

CeO_2 grain dynamics. By monitoring the evolution of different grains on the sample (Figure S19A and B), we have found that smaller CeO_2 grains with different orientations combine into larger grains. For example, the grain colored with green in the middle bottom of the nanorods (A) is completely fused into the grain colored with red at high temperature (B). The crystallinity of these grains also improves as temperature rises, as the (111) diffractions from CeO_2 lattices at

low temperature becomes sharper and dominant with increasing temperature (Figure S14C).

References

1. Dai, Y.; Lim, B.; Yang, Y.; Cobley, C. M.; Li, W.; Cho, E. C.; Grayson, B.; Fanson, P. T.; Campbell, C. T.; Sun, Y.; Xia, Y. *Angew. Chem. Int. Ed.* **2010**, *49*, 8165-8168.
2. Zhou, H.; Wu, H.; Shen, J.; Yin, A.; Sun, L.; Yan, C. *J. Am. Chem. Soc.* **2010**, *132*, 4998-4999.
3. Chen, C.; Fang, X.; Wu, B.; Huang, L.; Zheng, N. *ChemCatChem* **2012**, *4*, 1578-1586.
4. Yoon, K.; Yang, Y.; Lu, P.; Wan, D.; Peng, H.; Stamm Masias, K.; Fanson, P. T.; Campbell, C. T.; Xia, Y. *Angew. Chem. Int. Ed.* **2012**, *51*, 9543-9546.
5. Lu, P.; Campbell, C. T.; Xia, Y. *Nano Lett.* **2013**, *13*, 4957-4962.
6. Wang, X.; Liu, D.; Song, S.; Zhang, H. *J. Am. Chem. Soc.* **2013**, *135*, 15864-15872.
7. Nguyen, T. D.; Do, T. O. *J. Phys. Chem. C* **2009**, *113*, 11204-11214.
8. Xiao, W.; Guo, Q.; Wang, E. *Chem. Phys. Lett.* **2003**, *368*, 527-531.
9. Deshpande, S.; Patil, S.; Kuchibhatla, S. V.; Seal, S. *Appl. Phys. Lett.* **2005**, *87*, 133113.
10. McCafferty, E.; Wightman, J. P. *Surf. Interface Anal.* **1998**, *26*, 549-564.
11. Halder, A.; Sharma, S.; Hegde, M. S.; Ravishankar, N. *J. Phys. Chem. C* **2009**, *113*, 1466-1473.
12. Mao, M.; Lv, H.; Li, Y.; Yang, Y.; Zeng, M.; Li, N.; Zhao, X. *ACS Catal.* **2016**, *6*, 418-427.
13. Bera, P.; Priolkar, K. R.; Gayen, A.; Sarode, P. R.; Hegde, M. S.; Emura, S.; Kumashiro, R.; Jayaram, V.; Subbanna, G. N. *Chem. Mater.* **2003**, *15*, 2049-2060.
14. Li, C.; Sakata, Y.; Arai, T.; Domen, K.; Maruya, K.; Onishi, T. *J. Chem. Soc., Faraday Trans. 1* **1989**, *85*, 929-943.
15. Li, C.; Sakata, Y.; Arai, T.; Domen, K.; Maruya, K.; Onishi, T. *J. Chem. Soc., Faraday Trans. 1* **1989**, *85*, 1451-1461.
16. Socrates, G., *Infrared and Raman Characteristic Group Frequencies: Tables and Charts*. Wiley: 2001.
